# SDF-1/CXCR4 axis induces human dental pulp stem cell migration through FAK/PI3K/Akt and GSK3β/β-catenin pathways

**DOI:** 10.1038/srep40161

**Published:** 2017-01-09

**Authors:** Mingwei Li, Xuefei Sun, Liang Ma, Lu Jin, Wenfei Zhang, Min Xiao, Qing Yu

**Affiliations:** 1 State Key Laboratory of Military Stomatology & National Clinical Research Center for Oral Diseases & Shaanxi Key Laboratory of Stomatology, Department of Operative Dentistry and Endodontics, School of Stomatology, The Fourth Military Medical University, Xi’an 710032, People’s Republic of China; 2 Translational Research Team, School of Stomatology, The Fourth Military Medical University, Xi’an 710032, People’s Republic of China; 3 State Key Laboratory of Military Stomatology & National Clinical Research Center for Oral Diseases & Shaanxi Key Laboratory of Stomatology, Medical Plastic and Aesthetic Center, School of Stomatology, The Fourth Military Medical University, Xi’an 710032, People’s Republic of China

## Abstract

SDF-1 (stromal cell derived factor-1) has been found to be widely expressed during dental pulp inflammation, while hDPSCs (human dental pulp stem cells) contribute to the repair of dental pulp. We showed that the migration of hDPSCs was induced by SDF-1 in a concentration-dependent manner and could be inhibited with siCXCR4 (C-X-C chemokine receptor type 4) and siCDC42 (cell division control protein 42), as well as drug inhibitors such as AMD3100 (antagonist of CXCR4), LY294002 (inhibitor of PI3K) and PF573228 (inhibitor of FAK). It was also confirmed that SDF-1 regulated the phosphorylation of FAK (focal adhesion kinases) on cell membranes and the translocation of β-catenin into the cell nucleus. Subsequent experiments confirmed that the expression of CXCR4 and β-catenin and the phosphorylation of FAK, PI3K (phosphoinositide 3-kinase), Akt and GSK3β (glycogen synthase kinase-3β) were altered significantly with SDF-1 stimulation. FAK and PI3K worked in coordination during this process. Our findings provide direct evidence that SDF-1/CXCR4 axis induces hDPSCs migration through FAK/PI3K/Akt and GSK3β/β-catenin pathways, implicating a novel mechanism of dental pulp repair and a possible application of SDF-1 for the treatment of pulpitis.

Dental caries, the major cause of dentin damage, are widely seen worldwide. At the late stage of caries, inflammation will emerge at the dental pulp under the caries and induce apoptosis of odontoblasts. This process can be partially repaired under the effect of human dental pulp stem cells (hDPSCs). HDPSCs belong to mesenchymal stromal cells (MSCs) and express a number of chemokine and adhesion receptors^1^. Maintained in the stem cell niche around the blood vessels^2^, hDPSCs can be stimulated by chemokines expressed in extracellular matrixes. Thereafter, hDPSCs migrate to the damaged area and play a vital role during the regeneration of odontoblasts and the formation of reparative dentin^3^.

Chemotaxis is a complex cell behavior initiated by the binding of chemokines to their receptors, and accompanied by rearrangement of the cytoskeleton^4^. Chemokines are a group of secreted proteins able to induce the migration of cells that highly express their receptors^5^. Stromal cell derived factor-1 (also known as SDF-1 or CXCL12) interacts with its G-protein coupled receptor CXCR4 to induce SDF-1/CXCR4 signaling^6^. SDF-1 has also been shown to bind CXCR7^7^, but this binding does not contribute significantly to cell chemotaxis^8^. Ischemic kidneys could recruit MSCs by secreting SDF-1, which could be blocked by an antibody against CXCR4 but not against CXCR7^9^. Under physiological conditions, SDF-1/CXCR4 interaction participates in hematopoiesis and vascular development^10^. SDF-1 is essential for the migration of hematopoietic stem cells between bone marrow and blood^11^. The concentration of SDF-1 significantly correlates its effects, and excessive concentrations may act as an inhibitor^12^. Under pathological conditions, SDF-1 also contributes to the invasion and metastases of several tumor cells^13^. CXCR4 is expressed in a number of cancer cells, including prostate cancer, breast cancer and lung cancer. Additionally, inhibiting the activity of SDF-1 or blocking its binding to CXCR4 could reduce the migration or metastasis of cancer cells^14^. The SDF-1/CXCR4 axis is also upregulated during experimental models of burn wounds or myocardial infarction (MI)^15^.

Previous studies have shown that inflammation could improve the expression level of stromal cell derived factor-1α in dental pulp^16^. However, the signal networks that connect SDF-1 with the cell migration are still not well understood. The phosphoinositide 3-kinase (PI3K)/protein kinase B (Akt) activation has been shown to participate in the chemotaxis of MSCs^17^. AMD3100 could reverse the levels of phospho-Akt during MSCs migration induced by SDF-1/CXCR4 axis. Inhibiting the activation of glycogen synthase kinase-3β (GSK3β) could increase the expression of CXCR4^18^. β-catenin, which mediates the generation of mesoderm during embryogenesis^19^, participates in the migration and invasiveness of human mesenchymal cells^20^. It locates at the cell surface and links to actin cytoskeleton by binding to α-catenin. Upon activation, β-catenin dissociates from the cell membrane and translocates into nucleus to regulate the expressions of cytoskeleton proteins^21^. Previous reports demonstrated that GSK3β inhibition could augment the expression of β-catenin^22^.

During the process of chemotaxis, CDC42 which locates at the leading edge could be activated^23^. CDC42 belongs to the Rho GTPases family which regulates the arrangement of actin cytoskeleton and affects cell motility^24^. The dynamic remodeling of the cytoskeleton is necessary for cell migration. CDC42 modulates cell migration by coordinating the actin-based structures located at the leading edge of cells^25^. It is generally thought that CDC42 induce the formation of cellular protrusions such as filopodia by activating actin-associated proteins and inducing F-actin bundles^26^. Those actin-associated proteins include Wasp (Wiskott-Aldrich syndrome protein) and PAKs (p21-activated kinases), while Wasp regulates the formation of filopodia and actin polymerization and PAKs affects the activation of actin-binding protein cofilin^27^.

In the present study, we investigated the ability of SDF-1 to increase the migration of hDPSCs. Considering that both PI3K/Akt and GSK3β/β-catenin play roles during cell migration, we hypothesized that SDF-1 may regulate the migration of hDPSCs through these pathways. Additionally, given that CDC42 plays a critical role in cytoskeleton rearrangements, we hypothesized that the migration of hDPSCs stimulated by SDF-1 requires the participation of CDC42.

## Results

### The expression of CXCR4 and SDF-1 and their role in promoting migration in human dental pulp stem cells

**Figure 1:**
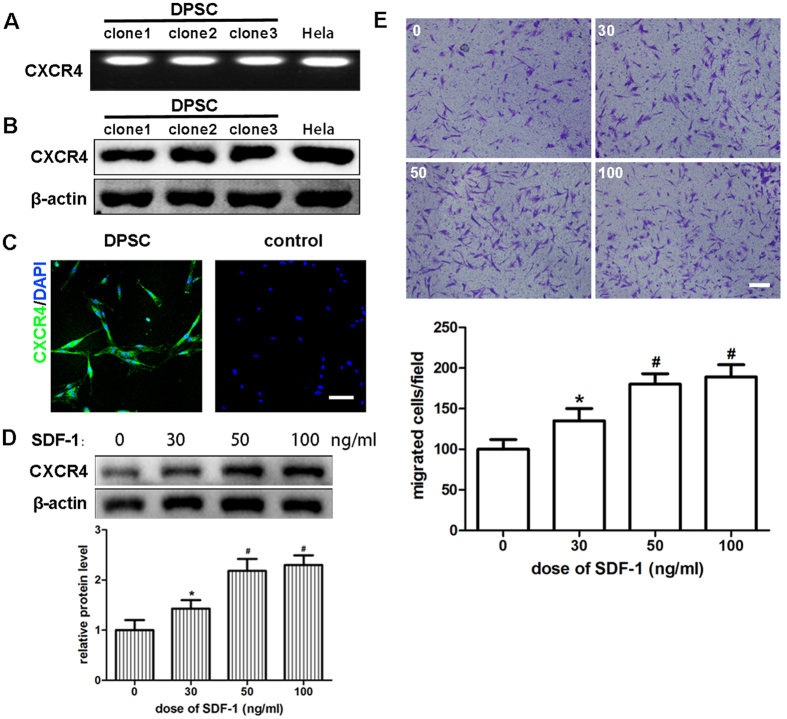
Expression of CXCR4 and SDF-1 and their role in promoting migration of hDPSCs. (**A**) RT-PCR analysis of CXCR4 mRNA in hDPSCs. (**B**) CXCR4 was identified by Western blot in hDPSCs. HeLa cells (**A** and **B**) were taken as positive control. (**C**) Immunofluorescence analysis of CXCR4 expression in hDPSCs. Cells not incubated with primary antibodies were taken as negative control. (**D**) The hDPSCs were cultured in medium with the indicated dose of SDF-1 for 48 hours and subjected to Western blot using CXCR4 antibody. (**E**) The hDPSCs were used in Transwell assays with the indicated dose of SDF-1 in the lower well. Cells that migrated to the lower side of the well were fixed and stained for 12 hours. Representative images are shown. The quantitative results (**D** and **E**) are the means ± SD of three independent experiments. Comparison with the control group (0 ng/ml): *p < 0.05; ^#^p < 0.01. In (**C**), scale bar = 100 μm; in (**E**), scale bar = 200 μm.

Because SDF-1, as one type of chemokine, induces cell migration through its seven-transmembrane receptor CXCR4, we first analyzed the expression of CXCR4 in hDPSCs by RT-PCR, Western blot and immunofluorescence. Considering that CXCR4 had been well studied in HeLa cells, we chose them as the positive control. Because hDPSCs form clones during proliferation, six clones were divided into two groups to detect the expression of mRNA and protein. The results of electrophoresis showed CXCR4 mRNA expressions in all clones of hDPSCs as abundantly as that in HeLa (Fig. 1A). The expression levels of endogenous CXCR4 protein varied in these three clones, but all of them showed positive expression when compared with HeLa (Fig. 1B). The expression of CXCR4 in hDPSCs at the cell level was also observed by immunofluorescence. Cells incubated without a primary antibody were taken as the negative control (Fig. 1C). The Western blot results showed that SDF-1 affected the expression of CXCR4 in a dose-dependent manner (Fig. 1D).

We next detected the effect of SDF-1 on the migration of hDPSCs. Transwell assays were performed, and a concentration-dependent manner was obtained in this section (Fig. 1E). The increasing dose of SDF-1 in the lower compartment of Transwell significantly promoted the number of cells migrating to the lower side of the inserts. Even if the dose was as low as 30 ng/ml, SDF-1 treated cells still showed a difference compared with the control group. A statistically significant difference was observed when the dose was increased to 50 ng/ml.

### SDF-1 mediated concentration-dependent and time-dependent activation of FAK, PI3K, Akt, and GSK3β and expression of β-catenin

**Figure 2:**
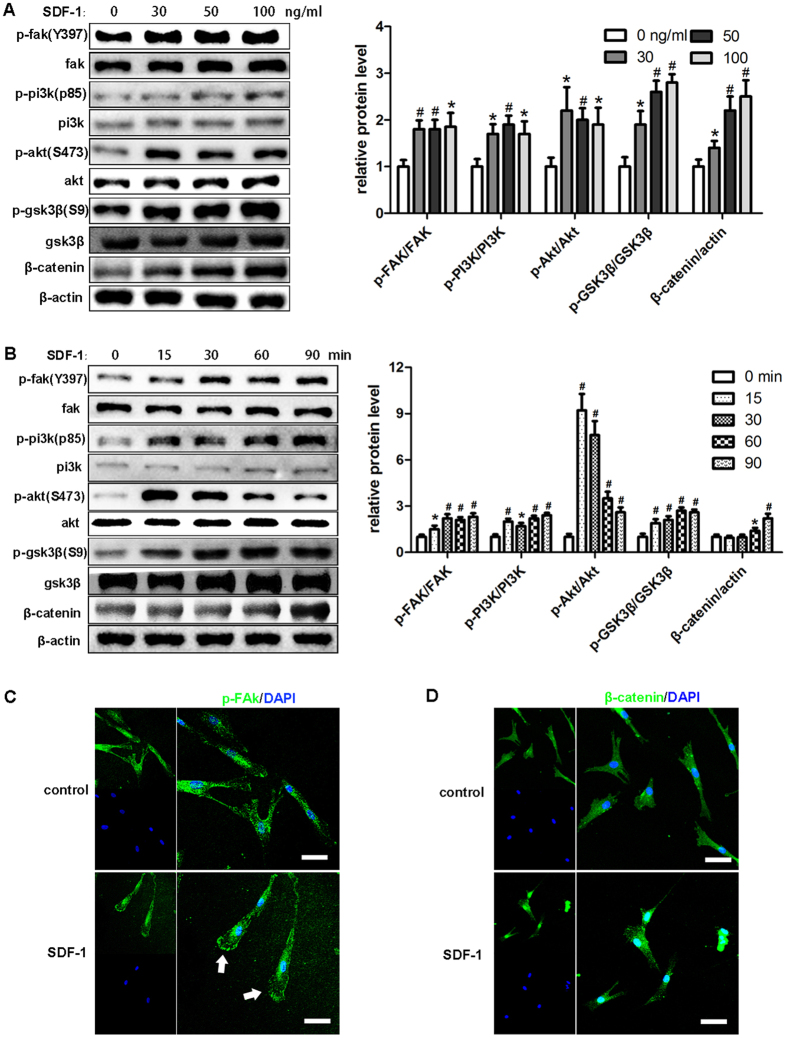
SDF-1 mediated concentration-dependent and time-dependent effects on FAK, PI3K, Akt, GSK3β and β-catenin protein expression. (**A**) The hDPSCs cultured in medium with the indicated dose of SDF-1 for 2 hours were subjected to Western blots. (**B**) The hDPSCs cultured in medium with SDF-1 (50 ng/ml) for the indicated times were detected by Western blot. Representative images and quantitative results (**A** and **B**) are shown. The membrane expression of p-FAK (**C**) and the nucleus translocation of β-catenin (**D**) were obtained after 50 ng/ml SDF-1 stimulation for 6 hours. Cells without SDF-1 stimulation were taken as control. The quantitative results (**A** and **B**) are the means ± SD of three independent experiments. The white arrows (**C**) indicate p-FAK at the leading edge. Comparison with the control group: *p < 0.05; ^#^p < 0.01. In (**C**) and (**D**), scale bars = 50 μm.

SDF-1 mediated expression and activation of CXCR4 has been shown to regulate several signaling pathways related to cell development and migration^28^. To investigate the signal transition induced by SDF-1 in hDPSCs, we examined the phosphorylated protein level changes of FAK, PI3K, Akt and GSK3β, as well as the protein level alterations of β-catenin after cells were stimulated by the indicated concentration of SDF-1 (30, 50, or 100 ng/ml) for 2 hours (Fig. 2A). Compared with the control group, cells treated with SDF-1 showed greater expression of phosphorylated proteins. More phosphorylated GSK3β and β-catenin were detected with increasing concentration of SDF-1. However, no obvious differences of the expression of the phosphorylated proteins FAK, PI3K and Akt were observed among the treatment groups. Next, time course experiments were performed to examine the phosphorylated protein level alteration over time (Fig. 2B). The results obtained at 15 min showed that all the level of phosphorylated proteins in the treatment group were higher level than in the control group. Especially, the phosphorylated Akt increased more than 8 folds. However, the phosphorylated Akt level was downregulated as time increased to 90 min. Meanwhile, the expressions of β-catenin did not show any increase until 60 min.

Considering that cell migration depends on the regulation of focal adhesions by FAK and cell skeleton rearrangement by β-catenin, we determined their distributions after SDF-1 stimulation for 6 hours in hDPSCs. The results obtained with p-FAK immunofluorescence showed that enhanced expression of phosphorylated FAK was found at the leading edge of migrating cells compared to the control group (Fig. 2C). However, under the same condition, higher levels of β-catenin were located in the nucleus where they regulated cell migration by acting as a transcription factor (Fig. 2D).

### AMD3100 suppresses SDF-1/CXCR4 signaling and hDPSCs migration

**Figure 3:**
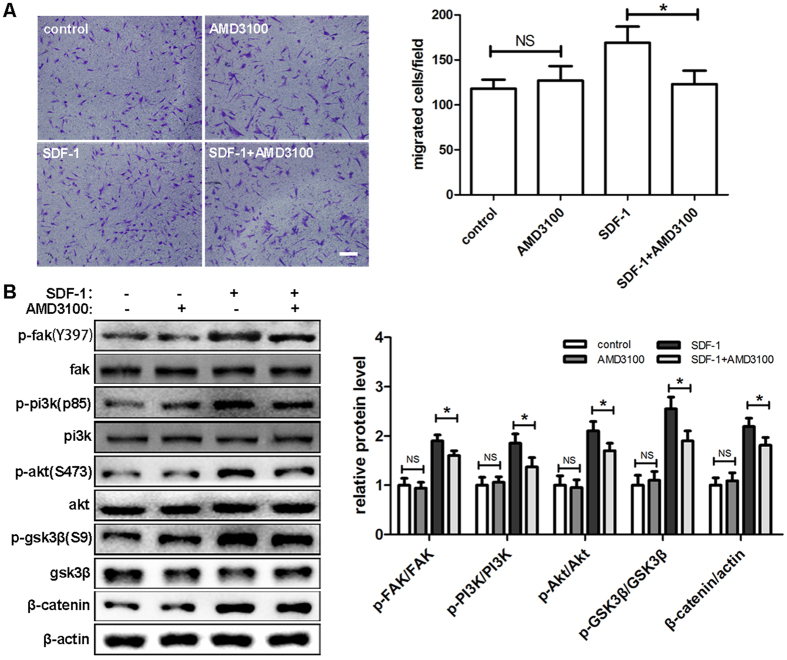
AMD3100 suppressed SDF-1/CXCR4 signaling and hDPSCs migration. (**A**) After preincubation with AMD3100 (100 ng/ml) for 1 hour, hDPSCs were used in Transwell assays with AMD3100 in the upper chamber and SDF-1 in the lower chamber and allowed to migrate for 12 hours. (**B**) After preincubation with AMD3100 for 1 hour and culture in medium with the indicated drug concentrations (100 ng/ml AMD3100, 50 ng/ml SDF-1) for 2 hours, cells were subjected to Western blots. The quantitative results (**A** and **B**) are the means ± SD of three independent experiments. NS, not significant; *p < 0.05. In (**A**), scale bar = 200 μm.

As a selective CXCR4 antagonist, AMD3100 could disrupt the interaction of SDF-1 and CXCR4^29^. Transwell assays were performed to observe its effects on hDPSCs migration. The results showed that AMD3100 significantly reduced cell migration caused by SDF-1, while no reduction was detected in the group treated with AMD3100 alone (Fig. 3A). Consistent with the migration study, no alteration was found in the level of phosphorylated protein of FAK, PI3K, Akt and GSK3β with AMD3100 treatment alone. However, AMD3100 successfully inhibited the increases of p-FAK, p-PI3K, p-Akt and p-GSK3β induced by SDF-1 treatment. The same results were also obtained in the expression of β-catenin (Fig. 3B).

### Downregulation of CXCR4 by siRNA transfection decreases hDPSCs migration and SDF-1/CXCR4 signaling

**Figure 4:**
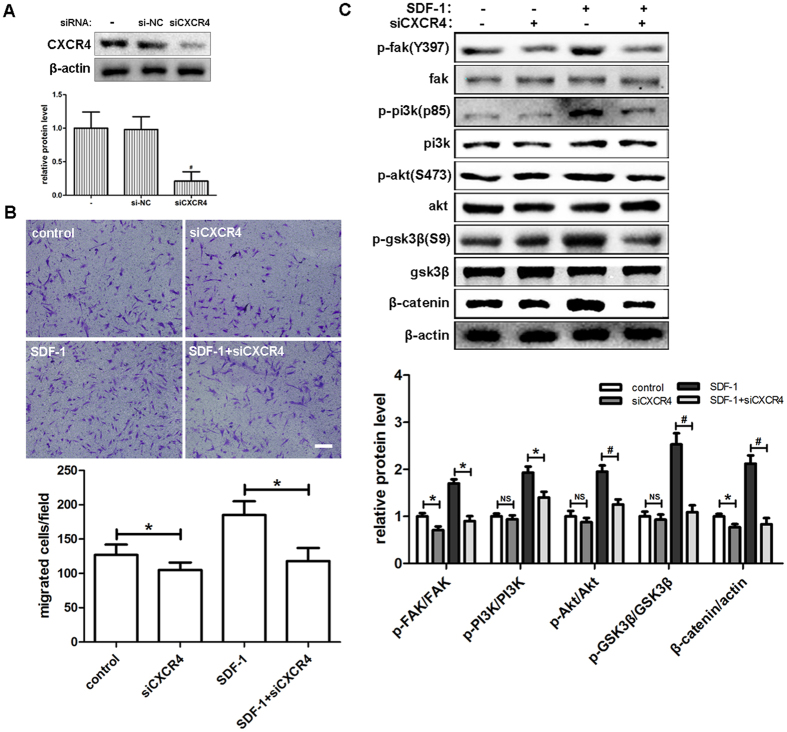
Downregulation of CXCR4 by siRNA transfection decreased the migration of hDPSCs and SDF-1/CXCR4 signaling. (**A**) After transfection with siCXCR4 for 48 hours, hDPSCs were subjected to Western blot and CXCR4 was detected with the indicated antibody. (**B**) Cells transfected with siCXCR4 were used in Transwell assays with the indicated medium (50 ng/ml SDF-1) in the lower chamber. Cells that migrated to the lower side of the well were fixed and stained for 12 hours. Representative images are shown. (**C**) After transfection with siCXCR4 for 48 hours, cells were cultured in the indicated medium (50 ng/ml SDF-1) for 2 hours and subjected to Western blots. The quantitative results (**A**,**B** and **C**) are the means ± SD of three independent experiments. NS, not significant; *p < 0.05; ^#^p < 0.01. In (**B**), scale bar = 200 μm.

Since the function of SDF-1 depends on the activation of CXCR4, we investigated whether downregulation of CXCR4 by siRNA transfection could affect the migration of hDPSCs as we had observed with AMD3100 inhibition. First, we confirmed the knockdown effect of siRNA on the expression of CXCR4 in hDPSCs (Fig. 4A). Unlike AMD3100, treatment with siCXCR4 alone also inhibited the migration of hDPSCs in the Transwell assays. The increases of migrated cells by SDF-1 were completely diminished by siCXCR4 treatment (Fig. 4B). The results obtained with Western blot were similar to those of the Transwell assays. The expression of p-FAK and β-catenin was decreased by siCXCR4 when compared with the control group. The level of p-PI3K, p-Akt, and p-GSK3β also showed a slight downregulation with siCXCR4 alone, although no statistically significant differences were detected. In groups treated with SDF-1, all the levels of phosphorylated proteins and β-catenin experienced a significant reduction when pretreated with CXCR4 siRNA (Fig. 4C).

### PI3K inhibition by LY294002 plays a negative role during the migration of hDPSCs

**Figure 5:**
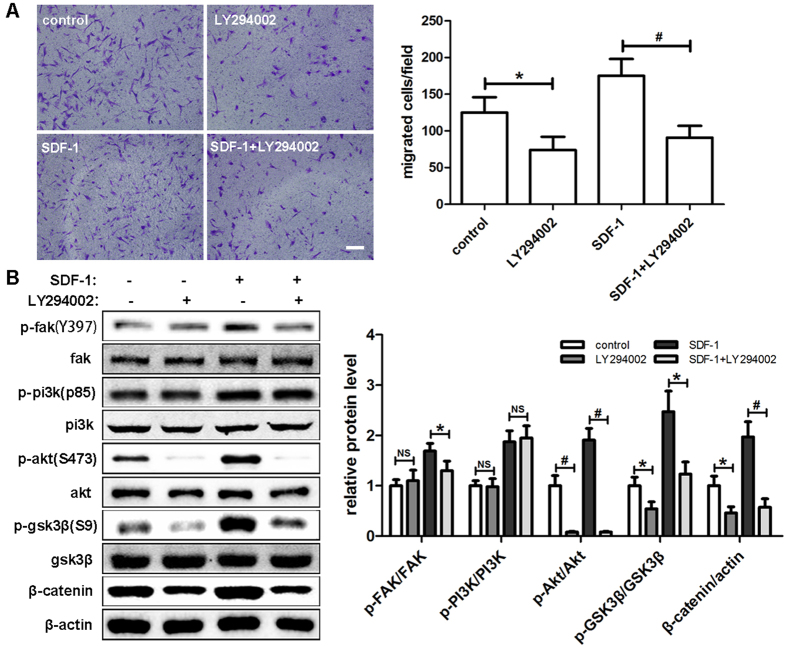
PI3K inhibition by LY294002 played a negative role during the migration of hDPSCs. (**A**) After preincubation with LY294002 (20 μM) for 1 hour, hDPSCs were used in Transwell assays with LY294002 in the upper chamber and SDF-1 in the lower chamber and allowed to migrate for 12 hours. (**B**) After preincubation with LY294002 for 1 hour and culture in medium with the indicated drug concentrations (20 μM LY294002, 50 ng/ml SDF-1) for 2 hours, cells were subjected to Western blots. The quantitative results (**A** and **B**) are the means ± SD of three independent experiments. NS, not significant; *p < 0.05; ^#^p < 0.01. In (**A**), scale bar = 200 μm.

To assess the effect of PI3K/Akt signaling pathway on the migration of hDPSCs, cells were pretreated with 20 μM LY294002, a specific PI3K inhibitor, for 1 hour before SDF-1 treatment. A clear reduction by approximately 30% in migrating cells was detected when cells were treated with LY294002 alone. This finding suggests that the PI3K/Akt signaling pathway is necessary for hDPSCs migration. When combined with SDF-1, LY294002 still showed a large inhibitory effect compared with groups treated with SDF-1 alone (Fig. 5A). In subsequent experiments, we examined the expression levels of phosphorylated proteins in hDPSCs (Fig. 5B). Compared with the control group, p-Akt showed an extraordinary downregulation of more than 90% after LY294002 treatment alone. Similar results were also obtained for p-GSK3β and β-catenin with reductions of approximately 50%, but no significant differences were detected with p-FAK and p-PI3K. When combined with SDF-1, LY294002 significantly downregulated the expression levels of p-FAK, which was different from that with LY294002 alone. The comparisons of the other protein expressions were similar to what we observed in groups without SDF-1 treatment.

### Effect of FAK inhibitor PF573228 on hDPSCs migration and SDF-1/CXCR4 signaling

**Figure 6:**
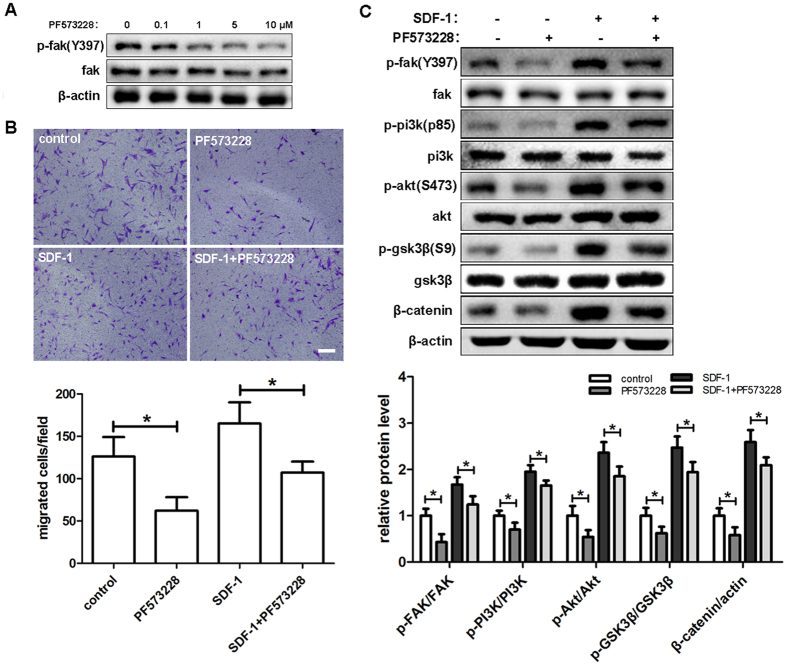
Effect of FAK inhibitor PF573228 on hDPSCs migration and SDF-1/CXCR4 signaling. (**A**) hDPSCs were cultured in medium with the indicated concentration of PF573228 for 2 hours and then subjected to Western blot using the indicated antibodies. (**B**) After preincubation with PF573228 (10 μM) for 1 hour, the hDPSCs were used in Transwell assays with LY294002 in the upper chamber and SDF-1 in the lower chamber and allowed to migrate for 12 hours. (**C**) After preincubation with PF573228 (10 μM) for 1 hour, hDPSCs were subjected to Western blots and detected with the indicated antibodies. The quantitative results (**B** and **C**) are the means ± SD of three independent experiments. NS, not significant; *p < 0.05. In (**B**), scale bar = 200 μm.

Considering that focal adhesion kinase (FAK) is located on the cell membrane and interacts with the extra cellular matrix through integrins^30^, we determined whether FAK played a biological role during the migration of hDPSCs induced by SDF-1. Concentration-response experiments were performed, and 10 μM PF573228 was chosen to inhibit the activation of FAK based upon Western blot results (Fig. 6A). After treatment with PF573228 alone, the migration of hDPSCs was inhibited more than 50% compared with the untreated group. Similarly, the inhibitor also decreased hDPSCs migration after treatment with SDF-1 (Fig. 6B). In subsequent experiments, we analyzed the effect of PF573228 at the protein level and observed similar results as with cell migration. Whether the cells were treated with SDF-1 or not, the expression levels of p-FAK, p-PI3K, p-Akt, p-GSK3β and β-catenin were significantly downregulated by the inhibitor treatment. However, the differences between the groups treated with SDF-1 were no different than those without SDF-1 treatment (Fig. 6C).

### siRNA mediated CDC42 inhibition decreases hDPSCs migration

**Figure 7:**
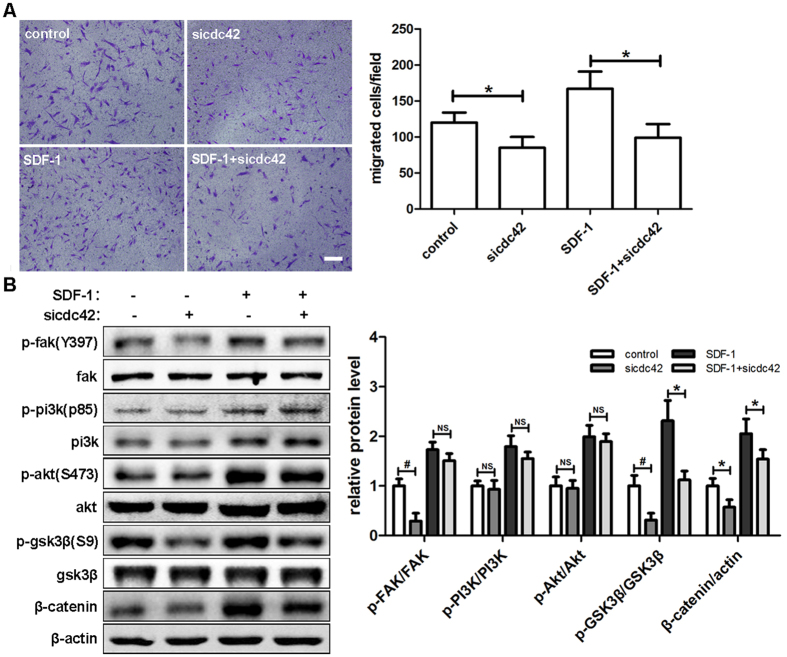
SiRNA mediated CDC42 inhibition decreased hDPSCs migration. (**A**) After transfection with siCDC42 for 48 hours, cells were used in Transwell assays with the indicated medium (50 ng/ml SDF-1) in the lower chamber and allowed to migrate for 12 hours. (**B**) After transfection with siCDC42 for 48 hours, cells were cultured in the indicated medium (50 ng/ml SDF-1) for 2 hours and then subjected to Western blots. The quantitative results (**A** and **B**) are the means ± SD of three independent experiments. NS, not significant; *p < 0.05; ^#^p < 0.01. In (**A**), scale bar = 200 μm.

Cell skeleton rearrangements are required during cell migration, and CDC42 plays a central role in this process. Thus, we hypothesized that the migration of hDPSCs induced by SDF-1 could be regulated by CDC42. After transfection with siCDC42 (unpublished results) for 48 hours, migration assays were performed and the migrating ability of hDPSCs was clearly inhibited. Combined with SDF-1, siCDC42 almost reversed the increase of hDPSCs migration triggered by SDF-1 alone (Fig. 7A). Next, the effects of siCDC42 on the protein expression of the SDF-1 signaling pathway were examined (Fig. 7B). When the cells were treated with siCDC42 alone, the expression levels of p-FAK, p-GSK3β and β-catenin were downregulated significantly. Similar results were obtained in the groups treated with SDF-1 and siCDC42, except that no significant difference was detected for p-FAK. As for the phosphorylation levels of PI3K and Akt, no differences were observed with siCDC42, whether the cells were treated with SDF-1 or not.

## Discussion

The development of dental caries typically comes along with the invasion of bacteria into dentin, and the induction of pulpitis occurs when the odontoblasts under the caries are infected by the bacteria. Pulpitis is characterized by the loss of dentin and destruction of odontoblast cell layers. After treatment, the repair of the damaged tissues under both situations requires the participation of hDPSCs. However, because the hDPSCs reside in the stem cell niches near the blood vessels in the dental pulp, it is required for them to expand and migrate to the damaged area to generate new odontoblasts for repair. The migration and homing of hDPSCs to damaged tissue are probably a multistep process and share some common characteristics with the migration of leukocyte to inflammatory sites^31^. Because hDPSCs are used for therapeutic purposes, it would be important to understand the process regulating their migration.

The migration of stem cells is regulated by a number of secreted molecules, including chemokines. SDF-1 has been well known as an essential mediator during the physiological development of tissues and pathogenesis of many diseases^32^. SDF-1 has six different splicing variants (α, β, γ, δ, ε, ψ), of which SDF-1α is expressed widely across different tissues and mediates several different activities, including adhesion, chemotaxis and survival^33^. All the isoforms share a same N-terminal domain to bind and activate receptors and a different C-terminal domain to stabilize the interactions with receptors^34^. Increasing the expression level of SDF-1 could increase the capillary density of the myocardium after MI^35^. As a stem cell homing factor, SDF-1 contributes to tissue regeneration and healing by recruiting CXCR4 positive progenitors to the damaged area. The investigation of cell therapy for self-repair found that CXCR4, the receptor of SDF-1, was an important regulator of the migration towards the regions of brain injury^36^. Cytokines secreted during inflammation, including TNF (tumor necrosis factor) and VEGF (vascular endothelial growth factor), could also induce the expression of CXCR4^37^.

Although previous studies have confirmed that SDF-1 is highly expressed during the inflammation of the dental pulp^38^, the specific roles of SDF-1 in the repair of the dental pulp still remains unclear. In the present study, we first demonstrated the expression of CXCR4 in hDPSCs compared with HeLa cells. SDF-1 has been shown to be a principal regulator of migration and mobilization of endothelial progenitor cells and hematopoietic stem cells^39^. Experiments *in vivo* found that MI in rats increased the level of SDF-1 that induced the migration of BMSCs^40^. Many experiments performed with tissue injury have confirmed the upregulation of the SDF-1/CXCR4 axis, such as burn wounds^41^, acute kidney injury^42^ and MI^43^. If SDF-1 signaling was disturbed, the recruitment of MSCs to the bone fractures and the repair would be inhibited^44^. All the studies above support the importance of the role of SDF-1/CXCR4 during stem cell migration. Our data also demonstrate that both the expression of CXCR4 and the migration of hDPSCs are highly upregulated by the indicated concentrations of SDF-1. Studies on the trans-endothelial migration of hematopoietic stem cells have revealed that SDF-1 is an important chemoattractant^45^, which could be the presumed role of SDF-1 in this study.

The SDF-1/CXCR4 axis has been shown to regulate cell migration through several signaling pathways^46^. PI3K is widely involved in the regulation of multiple stem cell migration by coordinating the function and localization of lipids through pleckstrin homology (PH) domains^47^, through which PI3K also recruits the protein kinase Akt. However, there are no related studies of the role of SDF-1α in hDPSCs migration. Microarray studies have demonstrated that SDF-1/CXCR4 axis inhibition may affect the GSK3β/β-catenin pathway^48^. To understand the molecular mechanisms underlying hDPSCs migration, both concentration-response and time course experiments were performed to evaluate the SDF-1 signaling pathway and showed that SDF-1 induces the phosphorylation of FAK, PI3K, Akt, and GSK3β, as well as the expression of β-catenin. This finding suggests that all of them participate in the function of SDF-1 during hDPSCs migration, though the exact connections among these proteins remain to be examined.

Consequently, because CXCR4 plays a key role in SDF-1/CXCR4 axis, we sought to determine the effect of CXCR4 knockdown on the migration of hDPSCs induced by SDF-1. Research on vascular progenitor migration has identified that CXCR4 directly participated in this process^49^, and preclinical studies have revealed that metastasis of cancer is directly mediated by CXCR4^50^. Several pathways have been associated with CXCR4 in cancer cells, such as Erk/p38 and PI3K/Akt/NFκB, whereas CXCR4 inhibition deactivated Akt without Erk revealing cell context-specific pathway regulation^51^. In our present study, when no SDF-1 was added, the knockdown of CXCR4 still reduced the phosphorylation of FAK and the expression of β-catenin. This result suggests that CXCR4, more than a SDF-1 receptor, may also act as a regulator of another pathway and regulate cell migration through β-catenin. Under the stimulation of SDF-1, inhibiting the expression of p-FAK, p-PI3K, p-Akt, p-GSK3β and β-catenin by siCXCR4 identified their downstream roles in CXCR4 signaling in hDPSCs migration induced by SDF-1.

Additionally, our results also show that as the antagonist of CXCR4, AMD3100 significantly inhibited hDPSCs migration, which indicates a crucial role of SDF-1/CXCR4 axis in hDPSCs migration, though controversial evidence shows that blocking CXCR4 with AMD3100 could decrease tissue inflammation and increase progenitor cell migration to injury sites^52^. A previous study has demonstrated that the MSCs contribute to burn wound closure, which could be attenuated by the application of AMD3100^41^. The above observation was consistent with studies in cancer cells, of which migration would be inhibited with AMD3100 treatment or CXCR4 knockdown^53^. Treatment with AMD3100 could also diminish the recruitment of bone marrow derived stem cells (BMDCs) after MI^54^.

The use of specific inhibitor of CXCR4 and CXCR4 expression silencing in hDPSCs with siRNA in this study confirmed that the effect of SDF-1 on hDPSCs migration was dependent on PI3K/Akt signaling. Previous studies have identified that PI3K is involved in multiple stem cell migration^40^. The same phenomenon has also been demonstrated in leukemic cell lines^55^ and neural precursor cells^56^. The increased MSC migration by SDF-1 and hypoxia were both mediated by PI3K/Akt pathway^57^, which could be significantly attenuated by PI3K inhibitor. Using specific chemical inhibitors to detected the role of PI3K/Akt, our data demonstrated that blockade of PI3K could remarkably suppress the migration hDPSCs, as well as the induction of SDF-1, which was consistent with the role of PI3K/Akt in T lymphocytes^58^ and neural stem cells^59^. Under both conditions with and without SDF-1, the inhibitor of PI3K significantly abrogated the expression of β-catenin and the phosphorylation of Akt and GSK3β, indicating that all of them were downstream effectors of SDF-1/CXCR4/PI3K. A previous study on cardiac stem/progenitor cells (CSPC) showed that SDF-1 affected GSK3β activity via the phosphorylation of Akt^60^ and the β-catenin levels in pancreatic islet cells could be stabilized by SDF-1 through inhibition of GSK3β activity^61^, which is consistent with our results. Although the phosphorylation of FAK was not affected by the inhibitor of PI3K without SDF-1, it was downregulated by the inhibitor under SDF-1 stimulation. Moreover, phosphorylation of FAK at Y397 could induce the activation and recruitment of PI3K^62^. However, the phosphorylation of PI3K was inhibited by the inhibitor of FAK under both circumstances. These controversial results suggest that FAK and PI3K may work in coordination with each other during the hDPSCs migration induced by SDF-1.

In response to extracellular signaling mediated by G protein coupled receptors or Intergrins, FAK participates in the cytoskeleton rearrangements and the cycle of focal adhesion. It has also been shown to contribute epithelial cell migration^63^ and play roles in adhesion by activating multiple signaling pathways. We then determined that CXCR4 interacted with FAK in response to SDF-1. Previous experiments showed that FAK could be activated by SDF-1 in hematopoietic cell lineages, as well as in breast cancer cells^64^. Studies in progenitor B cells demonstrated that the prolonged activation of FAK correlated with the increased responsiveness of pro-B cells to SDF-1^65^. Our data indicates that the phosphorylation of FAK is necessary for the migration of hDPSCs and that specific inhibition of FAK by inhibitor leads to the decreased activation of the PI3K/Akt pathway. These data remind us again that FAK works at the upstream of the PI3K/Akt pathway.

Previous evidence has identified that Rho family GTPases regulate cell migration by affecting cell-cell adhesion and cytoskeleton rearrangements^66^. During cell migration, CDC42 controls the formation of filopodia^67^, which may participate in the recognition of extracellular signals^68^, such as chemotactic gradients. SDF-1 could also induce actin polymerization and initiate signaling pathways involved in cytoskeleton rearrangement^69^. SDF-1α expression in liver cancer can activate small GTPases, including CDC42, through interacting with CXCR4^70^. Our results make it clear that CDC42 makes a difference during migration of hDPSCs induced by SDF-1. Although the PI3K/Akt pathway was not influenced by the knockdown of CDC42, both the upstream and downstream of PI3K/Akt was inhibited by siCDC42 during the migration of hDPSCs. We also noticed that the knockdown of CDC42 had little effect on the phosphorylation of FAK with SDF-1 stimulation, which may be the result of SDF-1 effect.

## Conclusion

**Figure 8:**
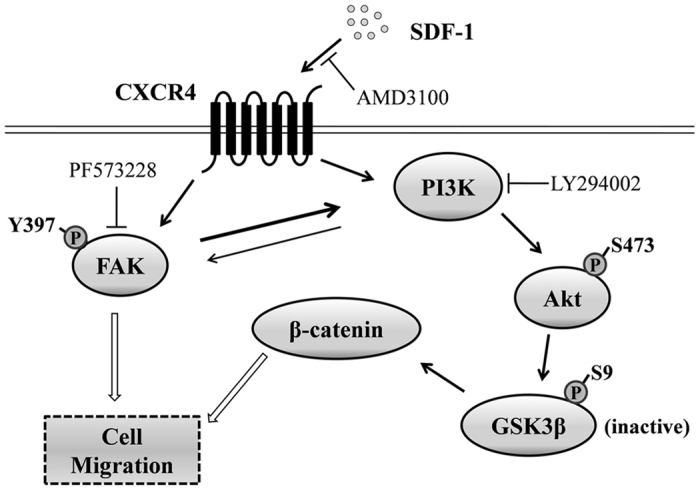
Schematic illustration showing the putative signaling pathways involved in SDF-1 mediated migration of hDPSCs. Extracellular SDF-1 induces the engagement of CXCR4, leading to the activation and coordination of FAK and PI3K. Consequently, the phosphorylation of Akt at ser473 inhibits the activation of GSK3β resulting in an increase of β-catenin expression. As a result, the SDF-1/CXCR4 axis activates FAK/PI3K/Akt and GSK3β/β-catenin pathways leading to the migration of hDPSCs.

Our studies delineate the mechanisms during the migration of hDPSCs towards SDF-1. SDF-1 is an important regulator of hDPSCs migration, though it remains to be elucidated whether it is secreted by stem cells or inflammatory cells. All the results above demonstrate that SDF-1/CXCR4 axis interacts with FAK/PI3K/Akt and GSK3β/β-catenin pathways in regulating hDPSCs migration (see Fig. 8 for overview). These studies provide a framework to study the mechanism of SDF-1 on hDPSCs migration and some directions on the application of SDF-1 in dental pulp repair. Further questions about the reactions of hDPSCs after SDF-1 application *in vivo* and how to use SDF-1 during treatment are the subjects of future investigation.

## Methods

### Isolation and culture of human dental pulp stem cells

Normal human third molars without caries were obtained from patients 18 to 22 years of age for orthodontic reasons or because of impaction. The whole procedure was carried on using a protocol approved by the Institutional Review Board of the Fourth Military Medical University. We confirmed that all methods were performed in accordance with the relevant guidelines and informed consent was obtained from all subjects. Briefly, the extracted teeth were transported to the laboratory on ice within 2 hours of collection. The dental pulp were dissected out of the teeth, minced and digested with 3 mg/ml collagenase type I at 37 °C for 45 min. After washing three times with PBS, cells were suspended in the α-modification of Eagle’s Medium (α-MEM) with 15% fetal bovine serum, 100 units/ml penicillin and streptomycin, seeded at the density of 2 × 10^3^/well in 6-well plates and cultured at 37 °C in a 5% CO_2_ incubator. The medium was refreshed every 2 days, and cells were passaged when reaching 80% confluence. Passages 3 to 5 were used in this study, and all experiments were performed at least three times.

### Immunofluorescence staining

Human dental pulp stem cells were seeded on coverslips overnight and fixed in 4% paraformaldehyde solution in PBS for 15 min at 4 °C. After washing with PBS three times, cells were permeated with 0.1% Triton-100 for 10 min and blocked by 5% bovine serum albumin (BSA) for 30 min at room temperature. Cells were then incubated at 4 °C with diluted anti-CXCR4 antibody (Abcam, 1:100) overnight. Fluorescein isothiocyanate (FITC) labeled donkey anti-rabbit secondary antibodies were added onto cells for 1 h at 4 °C after washing. Pictures were taken with Olympus FV1000 confocal microscope and acquired using the FV10-ASW3.1 Viewer software. After seeding for 12 hours and culture in α-MEM with 10% FBS and 50 ng/ml SDF-1α (Gibco, USA) for 2 hours, primary antibodies against phospho-FAK (Y397, Abcam) and β-catenin (Cell Signaling Technology) were used as described above. Dilutions without primary antibodies served as negative control.

### Western blot analysis

Human dental pulp stem cells were seeded in plates and reached 80% confluence at 37 °C in a 5% CO_2_ incubator. Cells were then washed with PBS and lysed in ice-cold radio-immunoprecipitation assay (RIPA) buffer with a complete protease inhibitor cocktail (Sigma). Primary antibodies against CXCR4, PI3K (p85α), phosphor-Akt (S473) and total Akt (Abcam), phospho-FAK (Y397), FAK, phospho-PI3K (p85), phospho-GSK3β (S9), GSK3β, β-catenin and β-ACTIN (Cell Signaling Technology) were purchased and used as recommended by the manufacturers. Proteins were extracted, resolved by SDS-PAGE and then transferred to PVDF (polyvinylidene difluoride) membranes. After blocking in 5% non-fat dry milk in TBST (Tris-Buffered Saline with Tween) for 2 hours and probing with the indicated primary antibodies at 4 °C overnight, the membranes were rinsed and incubated with dilutions of the appropriate secondary antibodies conjugated with horseradish peroxidase (Cell Signaling Technology) for 1 hour at room temperature and with the enhanced chemiluminescence kit (Millipore) for a few seconds. Signals were captured using ChemiDoc MP system (Bio-Rad) and Image Lab software (Bio-Rad). After treatment with SDF-1, siRNA or inhibitors for the indicated time at 37 °C in a 5% CO_2_ incubator, proteins were extracted and treated as described above. β-actin was used as an internal control. Quantization of the level of phosphorylated proteins was calculated by normalizing the p-form with the total amount.

### RT-PCR

Total cellular RNA was isolated from cells using Trizol reagent (Takara) according to the manufacturer’s instruction. The cDNA was synthesized using PrimeScript RT reagent Kit with gDNA Eraser (Takara) according to the manufacturer’s protocols. Standard PCR reactions were performed and the products were examined by electrophoresis on 1% agarose (Biowest, French) gel. After staining with EB (ethidium bromide) dilution, pictures were taken with the ChemiDoc MP system (Bio-Rad).

### siRNA Transfection

siCXCR4, siCDC42 and scrambled siRNA were designed and produced by RiboBio (Guangzhou, China). All siRNA were diluted to working concentration in RNase-free water. Human dental pulp stem cells were transfected with 50 nM siRNA using DharmaFECT Transfection Reagents (Dharmacon) according to manufacturer’s protocols. Briefly, cells were transfected with siRNA or vehicle when reaching 50–60% confluence. Total RNA was isolated after 24 hours. Total proteins were extracted and Transwell assays were performed after 48 hours.

### Cell Migration Assay using Transwell System

Human dental pulp stem cells were cultured in α-MEM with 10% FBS and penicillin/streptomycin to reach 50–60% confluence. For siRNA treatment, cells were transfected with siCXCR4 or siCDC42 as described above for 48 hours. For the inhibitor treatment, cells were preincubated with AMD3100 (100 ng/ml), LY294002 (20 μM) and/or PF573228 (10 μM) (all purchased from Sigma) for 1 hour at 37 °C in a 5% CO_2_ incubator. After treatment with the indicated siRNA or inhibitor in each group and dissociation with 0.25% trypsin into single cell suspension, cells were seeded in the upper chamber of a 24-well Transwell of 8 μm pore size (Corning, USA) with a density of 2.5 × 10^4^ cells/well and cultured in the same medium as that used before dissociation. Medium with SDF-1 (50 ng/ml) was added to the lower chamber. After migration at 37 °C for 12 hours, cells were washed with PBS, fixed with 4% paraformaldehyde solution in PBS for 15 min at 4 °C and stained with 0.1% crystal violet. On the upper side of the Transwell chamber, the cells were removed with a cotton applicator. On the lower side of the chamber, cells were counted in five random fields.

### Statistical Analysis

Data shown in this study are expressed as the mean ± standard deviation (SD) from at least three independent experiments. Statistical analysis was performed using SPSS Statistics 20.0 software. The statistical significance of the differences between two groups was analyzed by Student’s t test at a significance level of P < 0.05.

## Additional Information

**How to cite this article**: Li, M. *et al*. SDF-1/CXCR4 axis induces human dental pulp stem cell migration through FAK/PI3K/Akt and GSK3β/β-catenin pathways. *Sci. Rep.* **7**, 40161; doi: 10.1038/srep40161 (2017).

**Publisher's note:** Springer Nature remains neutral with regard to jurisdictional claims in published maps and institutional affiliations.
